# Supplementary material for: Central Dicer-miR-103/107 controls developmental switch of POMC progenitors into NPY neurons and impacts glucose homeostasis
Source: eLife. 2018 Oct 12;7:e40429. doi: 10.7554/eLife.40429 (PMC6203430; doi:10.7554/eLife.40429)
Supplement: Transparent reporting form [file elife-40429-transrepform.docx]

***eLife’s* transparent reporting form**

We encourage authors to provide detailed information *within their submission* to facilitate the interpretation and replication of experiments. Authors can upload supporting documentation to indicate the use of appropriate reporting guidelines for health-related research (see [EQUATOR Network](http://www.equator-network.org/%20)), life science research (see the [BioSharing Information Resource](https://biosharing.org/)), or the [ARRIVE guidelines](http://www.plosbiology.org/article/info:doi/10.1371/journal.pbio.1000412) for reporting work involving animal research. Where applicable, authors should refer to any relevant reporting standards documents in this form.

If you have any questions, please consult our Journal Policies and/or contact us: [editorial@elifesciences.org](mailto:editorial@elifesciences.org).

**Sample-size estimation**

- You should state whether an appropriate sample size was computed when the study was being designed
- You should state the statistical method of sample size computation and any required assumptions
- If no explicit power analysis was used, you should describe how you decided what sample (replicate) size (number) to use

Please outline where this information can be found within the submission (e.g., sections or figure legends), or explain why this information doesn’t apply to your submission:

We based our sample size on our 15+ years of experience with histochemical/neuroanatomical and physiological studies. We estimated that the proposed numbers of animals ensure statistical confidence in the results presented in the paper while reducing the number of animals used to meet scientific objectives.

**Replicates**

- You should report how often each experiment was performed
- You should include a definition of biological versus technical replication
- The data obtained should be provided and sufficient information should be provided to indicate the number of independent biological and/or technical replicates
- If you encountered any outliers, you should describe how these were handled
- Criteria for exclusion/inclusion of data should be clearly stated
- High-throughput sequence data should be uploaded before submission, with a private link for reviewers provided (these are available from both GEO and ArrayExpress)

Please outline where this information can be found within the submission (e.g., sections or figure legends), or explain why this information doesn’t apply to your submission:

We always repeated experiments at least twice and/or from at least 2 distinct litters to avoid experiment or litter bias.

For each experiment, replicate information is found in Methods section and in Figure Legends as described below:

- Intra-embryonic surgeries: page 19, Figure 4 legends

- Hanging drop culture: pages 19, 20

- Cell sorting: page 20

- Microarray: page 21, 22

For each experiment, *n* number information is found in Methods section and in Figure Legends as described below:

- Hanging drop: pages 19, 20 Figure 4 legends

- Tissue collection: page 20

- Cell sorting: page 20, Figure 1, Figure S3 legends

- Microarray: page 21, 22

- Tissue: page 22

- ISH: Figure2, Figure S2 legends

- Physiology: Pages 24, 25, Figure 1, Figure S1 legends

- Immunohistochemistry: Figure 1, Figure S2 legends

- Cell sorting: Figure 4 legends

- *In vitro* cultures: Figure 4 legends

Significant outliers were excluded based on Grubbs' test, also called the ESD method (Extreme Studentized Deviate), to determine whether one of the values was a significant outlier from the rest.

**Statistical reporting**

- Statistical analysis methods should be described and justified
- Raw data should be presented in figures whenever informative to do so (typically when N per group is less than 10)
- For each experiment, you should identify the statistical tests used, exact values of N, definitions of center, methods of multiple test correction, and dispersion and precision measures (e.g., mean, median, SD, SEM, confidence intervals; and, for the major substantive results, a measure of effect size (e.g., Pearson's r, Cohen's d)
- Report exact p-values wherever possible alongside the summary statistics and 95% confidence intervals. These should be reported for all key questions and not only when the p-value is less than 0.05.

Please outline where this information can be found within the submission (e.g., sections or figure legends), or explain why this information doesn’t apply to your submission:

All values were represented as the mean +/- SEM. Statistical significance was determined using unpaired 2-tailed Student’s t test, 1-way ANOVA followed by Tukey *post hoc* test, 2-way ANOVA followed by the Bonferroni *post hoc* test, and linear regression when appropriate. *P* ≤ 0.05 was considered statistically significant. This information is found in the Methods section, page 23 and in each Figure legend. The unpaired t test compares the means of two unmatched groups, assuming that the values follow a Gaussian distribution. 1-way ANOVA test is use to determine if there any change between two or more independent groups. 2-way ANOVA test is used to determine if there any change between two or more independent groups that have been split into 2 independent variables. Linear regression compares 2 variables. One variable is considered to be an explanatory variable, and the other is considered to be a dependent variable.

(For large datasets, or papers with a very large number of statistical tests, you may upload a single table file with tests, Ns, etc., with reference to sections in the manuscript.)

**Group allocation**

- Indicate how samples were allocated into experimental groups (in the case of clinical studies, please specify allocation to treatment method); if randomization was used, please also state if restricted randomization was applied
- Indicate if masking was used during group allocation, data collection and/or data analysis

Please outline where this information can be found within the submission (e.g., sections or figure legends), or explain why this information doesn’t apply to your submission:

For each experiment, slides were numerically coded to obscure the analysis. Information found in the Methods section.

**Additional data files (“source data”)**

- We encourage you to upload relevant additional data files, such as numerical data that are represented as a graph in a figure, or as a summary table
- Where provided, these should be in the most useful format, and they can be uploaded as “Source data” files linked to a main figure or table
- Include model definition files including the full list of parameters used
- Include code used for data analysis (e.g., R, MatLab)
- Avoid stating that data files are “available upon request”

Please indicate the figures or tables for which source data files have been provided:

Raw data of miRNA microarrays have been uploaded and are available. Data are summarized in Table 1.
